# The Clinical Significance of Programmed Death-1, Regulatory T Cells and Myeloid Derived Suppressor Cells in Patients with Nontuberculous Mycobacteria-Lung Disease

**DOI:** 10.3390/jcm8050736

**Published:** 2019-05-23

**Authors:** Chin-Chung Shu, Sheng-Wei Pan, Jia-Yih Feng, Jann-Yuan Wang, Yu-Jiun Chan, Chong-Jen Yu, Wei-Juin Su

**Affiliations:** 1 Department of Internal Medicine, National Taiwan University Hospital, Taipei 100, Taiwan; ccshu139@ntu.edu.tw (C.-C.S.); jywang@ntu.edu.tw (J.-Y.W.); 2 College of Medicine, National Taiwan University, Taipei 100, Taiwan; 3 Department of Chest Medicine, Taipei Veterans General Hospital, Taipei 112, Taiwan; swpan2@vghtpe.gov.tw (S.-W.P.); peterofeng@gmail.com (J.-Y.F.); wjsu@vghtpe.gov.tw (W.-J.S.); 4 School of Medicine, National Yang-Ming University, Taipei 112, Taiwan; 5 Institute of Public Health, National Yang-Ming University, Taipei 112, Taiwan; 6 Division of Infectious Diseases, Department of Medicine, Taipei Veterans General Hospital, Taipei 112, Taiwan; yjchan@vghtpe.gov.tw; 7 Division of Microbiology, Department of Pathology and Laboratory Medicine, Taipei Veterans General Hospital, Taipei 112, Taiwan

**Keywords:** cytotoxic T-lymphocyte antigen-4, *Mycobacterium avium* complex, *Mycobacterium abscessus*, myeloid derived suppressor cells, nontuberculous mycobacteria, programmed death-1, regulatory T cells

## Abstract

Background: Increasing expression of programmed death-1 (PD-1) in patients with nontuberculous mycobacteria lung disease (NTM-LD) has been reported, but its role in clinical characteristics and outcomes remains unclear. Methods: We enrolled 96 participants, including 46 with *Mycobacterium avium* complex (MAC)-LD, 23 with *M. abscessus* (MAB)-LD, and 27 controls. We measured expressions of PD-1, cytotoxic T-lymphocyte antigen-4 (CTLA-4) and regulatory T (Treg) cells on CD4^+^ lymphocytes and myeloid-derived suppressor cells (MDSCs) and analyzed their association with clinical features and radiographic outcomes. Results: The percentage of PD-1 on CD4^+^(PD-1^+^CD4^+^) lymphocytes and MDSCs were higher in the MAC-LD group than the controls. There were no intergroup differences regarding CTLA-4^+^CD4^+^ lymphocytes. Higher PD-1^+^CD4^+^ lymphocytes were found in *M. intracellulare-* and *M. avium*-LD than in other MAC-LD. Positive sputum acid-fast stains and fibrocavitary radiographic lesions were correlated with elevated PD-1^+^CD4^+^ lymphocytes and Treg cells. The percentage of PD-1^+^CD4^+^ lymphocytes at the initial and 2 months of follow-up significantly predicted subsequent radiographic progression. Conclusion: As markers of immune tolerance, PD-1^+^CD4^+^ lymphocytes and MDSCs were higher in MAC-LD patients. The levels of PD-1^+^CD4^+^ and Treg cells were correlated with high mycobacteria bacilli burden in NTM-LD. Monitoring the expressions of PD-1^+^CD4^+^ lymphocytes may predict radiographic progression.

## 1. Introduction

Nontuberculous mycobacteria lung disease (NTM-LD) has become an important clinical concern [1,2] because the prevalence of NTM infection has increased over the last two decades, however, the etiology remains unclear [3,4]. Among patients with NTM infection, *Mycobacterium avium* complex (MAC) and *Mycobacterium abscessus* (MAB) are the most predominant pathogens in North America and East Asia, and are the two most frequently isolated species responsible for NTM-LD [4,5]. In fact, only around one-third of patients with positive sputum cultures for MAC and MAB clinically have the disease [6,7], which highlights the importance of individual vulnerability to NTM infection [8].

The immune status of NTM-infected patients can become compromised through complex host-pathogen interactions, and recent studies have reported that the immune status of peripheral blood mononuclear cells (PBMC) is suppressed by MAC [9,10]. The mechanism responsible for the attenuated PBMC responses in NTM-LD has yet to be fully elucidated. Previous studies have shown that the expression of programmed death-1 (PD-1), a negative co-receptor for T cell activation [11] is higher in patients with MAC-LD, and may play a role in decreasing host immunity [10]. In regard to other immune regulators, regulatory T (Treg) cells are a subset of CD4^+^ T cells that regulate the host response to infection [12] by inhibiting the effecter functions of CD4^+^ and CD8^+^ T cells [13]. In addition, myeloid suppressor cells (MDSCs) are a group of undifferentiated immature innate cells with the ability to suppress T-cell responses [14]. However, the roles of the above immune regulators on host vulnerability and clinical manifestation have rarely been reported.

Although the course of NTM-LD is indolent, it has been associated with a decline in lung function [15] and a poor prognosis [16,17]. Approximately 22% and 53% of patients with MAC-LD have been reported to present with radiographic deterioration after 5 and 10 years, respectively [16]. However, there are currently no good predictors of the clinical outcomes of NTM-LD. Hence, in order to identify the subgroups with NTM-LD at risk of worse outcomes as early as possible, it is important to study the associations between immune regulators and different clinical presentations as well as radiographic progression.

## 2. Methods

### 2.1. Patient Enrollment

This prospective study was conducted at National Taiwan University Hospital (NTUH) from January 2014 to August 2017 and at Taipei Veterans General Hospital (TVGH) from January 2016 to August 2017. Patients aged ≥ 20 years and with at least two MAC- or MAB-positive sputum samples were assessed for NTM-LD according to the diagnostic guidelines suggested by the American Thoracic Society (Supplemental file) [1]. Patients with NTM-LD, including MAC-LD and MAB-LD, were recruited consecutively when they visited our chest or infection clinics. Patients with human immunodeficiency virus infection and active cancer were excluded. All participants in the healthy control group had negative chest radiographic images or were sputum-negative for NTM and had no major underlying disease.

The Research Ethics Committee of National Taiwan University Hospital (IRB No. 201407079RIND and 201512002RINC) and Taipei Veterans General Hospital (IRB No. 2014-09-008BC) approved this study. All of the participants provided written informed consent.

### 2.2. Isolation of Peripheral Blood Mononuclear Cells (PBMCs)

Peripheral blood from the participants was sampled into heparin-containing tubes. Mononuclear cells were immediately isolated using Ficoll-Paque PLUS (GE Healthcare Life Sciences, Uppsala, Sweden), and were then suspended in medium containing RPMI-1640 (Life Technologies, Carlsbad, CA, USA), 10% fetal bovine serum (FBS), and 1% penicillin-streptomycin (Life Technologies, Carlsbad, CA, USA).

### 2.3. Flow Cytometry of PBMCs

The PBMCs were stained for CD4, PD-1 and CTLA-4, and then measured using flow cytometry (FACSVerse, BD Biosciences, San Jose, CA, USA). We discriminated the lymphocyte population using forward scatter and side scatter. Within groups, we gated the subgroups of CD4^+^ T lymphocytes and measured the expressions of PD-1, and CTLA-4. The gating cut-off values were based on isotype staining. We also stained the PBMCs for CD4, CD25, and Foxp3, and defined positive staining for all three as showing Treg cells in the lymphocyte population. We measured CD3-/CD14-/HLA-DR- cells and then gated CD11b^+^/CD33^+^ cells to represent MDSCs using a modified protocol [18].

The staining antibodies were anti-CD4-APC, anti-CD25-FITC, anti-Foxp3-PerCP antibodies (BD Biosciences, San Jose, CA, USA), anti-PD-1-PE, and anti-CTLA-4-PEcy7.0 (eBiosciences, San Diego, CA, USA). Data were analyzed using BD FACSuite V software (BD Biosciences, San Jose, CA, USA).

### 2.4. NTM Species Identification

The NTM species were identified using conventional biochemical methods (at NTUH) or a molecular diagnostic biochip (at TVGH), as previously described [19]. To identify the subspecies of MAC, deoxyribonucleic acid of MAC isolates was extracted and subjected to polymerase chain reaction (PCR) targeting the β-subunit of RNA polymerase (rpoB) and the internal transcribed spacer (ITS) gene. The sequences of PCR products were checked, and the resultant MAC isolates were classified as *Mycobacterium avium*, *M. intracellulare*, *M. chimaera* and others [20].

### 2.5. Data Collection

Clinical data, radiographic findings and laboratory data at enrollment were recorded. Chest imaging was interpreted by radiographic score [21] and radiographic patterns of fibro-cavitary (FC), nodular bronchiectasis (NB), or others as previously reported [17]. Radiographic progression was defined as an increased score or new lesion after the initial year. Grading of sputum acid-fast bacilli staining (AFS), results of mycobacterium culture, and treatment for NTM-LD were recorded.

### 2.6. Statistical Analysis

Inter-group differences were analyzed using the Mann-Whitney *U* test for numerical variables, and Fisher’s exact test was used for categorical variables. Statistical significance was set at *p* < 0.05 in univariate analysis. Multivariate logistic regression analysis was used for adjusted odds ratios (ORs). The factors of age, sex, smoking and underlying disease were included and multivariate logistic regression was performed using stepwise methods. All analyses were performed using SPSS version 19.0 (Chicago, IL, USA).

## 3. Results

### 3.1. The Demographics of All Participants

A total of 96 participants were enrolled, including 46 patients with MAC-LD, 23 patients with MAB-LD, and 27 controls. The mean age of the patients with MAC-LD was 64.5 years, 44% were male, and the mean BMI was 20.0 kg/m^2^ (Table 1). In comparison, the controls were significantly younger (55.5 years) and had a higher BMI. With regards to the microbiological and radiographic features, the patients with MAC-LD had a higher sputum AFS (1.1 vs. 0.4, *p* = 0.020) and radiographic score (4.5 vs. 3.0, *p* = 0.020) than those with MAB-LD. There were no significant differences in the radiographic FC pattern and NB pattern between the MAC-LD and MAB-LD groups.

### 3.2. PD-1^+^CD4^+^ Lymphocytes, Treg Cells, and MDSCs in All Participants

**Figure 1:**
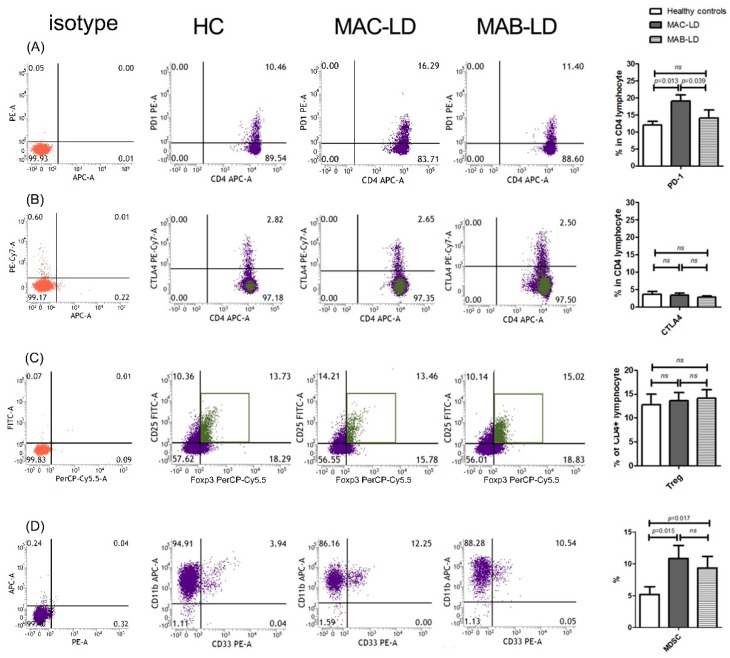
The proportion of (**A**) programmed death-1 (PD-1), (**B**) cytotoxic T-lymphocyte antigen-4 (CTLA-4), and (**C**) regulatory lymphocytes (Treg) in CD4 lymphocytes; (**D**) myeloid derived suppressor cells (MDSCs) according to the status of mycobacterial lung disease. The results shown are case demonstration and bar charts between the controls and patients with *Mycobacterium avium* complex-lung disease (MAC-LD) and those with *Mycobacterium abscessus*-LD (MAB-LD). We discriminated the lymphocytes and monocytes by forward scatter (FSC) and side scatter (SSC). We first gated the lymphocyte marker CD4, and then gated PD-1, CTLA-4, and CD25^+^/Foxp3^+^ in the CD4-positive lymphocytes. In Figure 1D, we gated CD3-/CD14-/HLA-DR- cells in peripheral blood mononuclear cells and then gated the CD11b^+^/CD33^+^ population. The data in the bar charts are mean values, and error bars are standard errors. The data were compared using the Mann Whitney U test. ns, not statistically significant (*p* > 0.05).

The percentage of PD-1 on CD4^+^ (PD-1^+^CD4^+^) lymphocytes was higher (19.1 ± 9.8%) in the patients with MAC-LD than in the controls (12.0 ± 4.6%, *p* = 0.013) and in those with MAB-LD (14.1 ± 9.3%, *p* = 0.039) (Figure 1A). The percentage of PD-1^+^CD4^+^ T cells was correlated with MAC-LD compared to the controls (OR: 1.133, 95% CI: 1.006–1.276, per 1% increment) after adjusting age and sex. In contrast, the percentage of CTLA-4 on CD4^+^ (CTLA-4^+^CD4^+^) lymphocytes was not significantly different among the three groups. The percentage of Treg cells, defined as CD4^+^CD25^+^Foxp3^+^, was not different among the three groups (Figure 1B). The proportion of MDSCs was higher in the MAC-LD (10.9 ± 10.6%) and MAB-LD (9.4 ± 8.1%) groups than in the controls (5.2 ± 5.1%, *p* = 0.015 and 0.017, respectively) (Figure 1C).

### 3.3. Differences among Different MAC Subspecies

**Figure 2:**
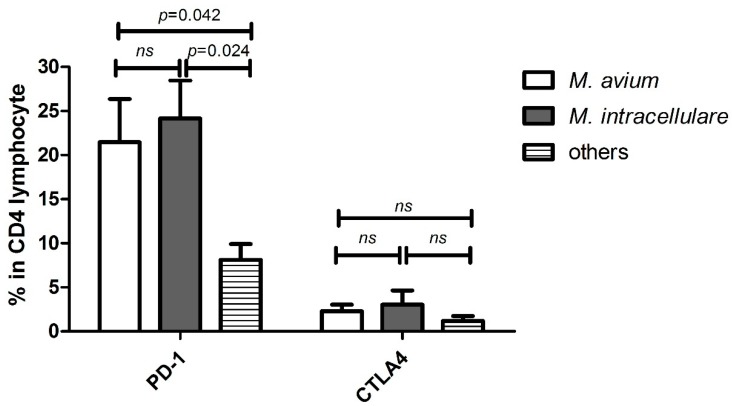
The proportion of programmed death-1 (PD-1) and cytotoxic T-lymphocyte antigen-4 (CTLA-4) on CD4^+^ lymphocytes in *Mycobacterium avium* complex-lung disease according to different causative subspecies. The data in the bar charts are mean values, and error bars are standard errors. The data were compared using the Mann Whitney U test. *M*., *mycobacterium*; ns, not statistically significant (*p* > 0.05).

We successfully retrieved 23 responsible strains from MAC-LD patients, and identified their MAC subspecies as *M. avium* (*n* = 10, 44%), *M. intracellulare* (*n* = 9, 39%), and others (*n* = 4, 17%; three with *M. chimera* and one with *M. timonense*). The percentages of PD-1^+^CD4^+^ lymphocytes were higher in the patients with MAC-LD caused by *M. avium* (21.5 ± 13.0%) and *M. intracellulare* (24.2 ± 11.1%) compared to the patients with MAC-LD caused by other subspecies (8.1 ± 3.6%, *p* = 0.042 and 0.024, respectively) (Figure 2). There were no significant differences in the percentages of CTLA-4^+^CD4 ^+^ lymphocytes, Treg cells and MDSCs among the subspecies.

### 3.4. The Influence of Mycobacteria Load on Immune Exhaustion

**Figure 3:**
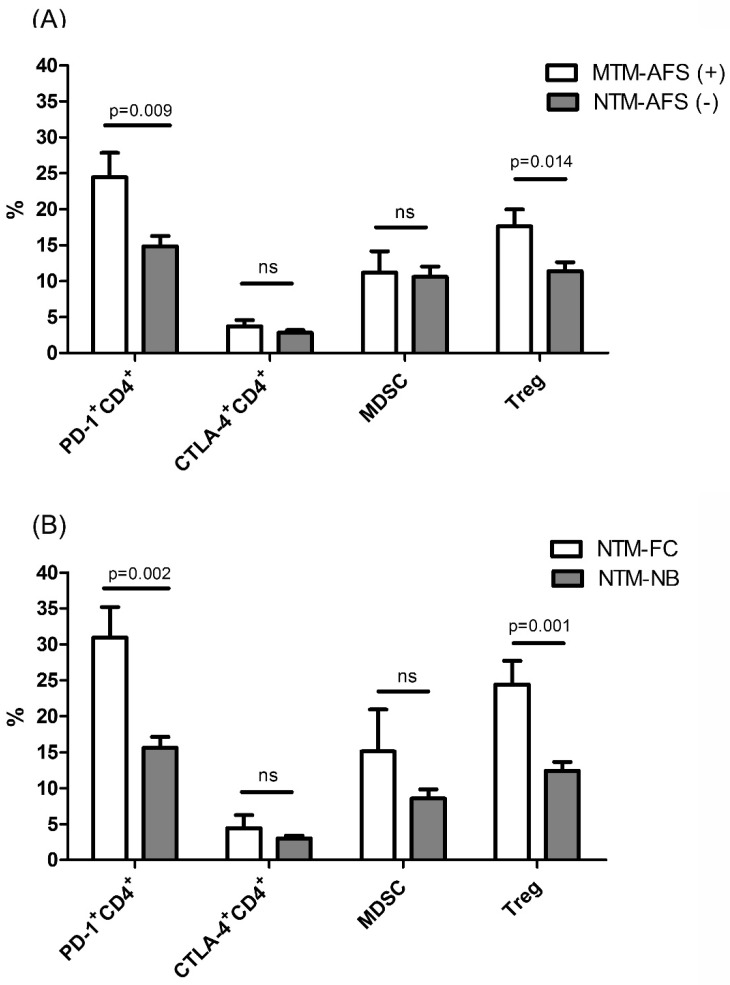
The percentages of regulatory T cells (Treg), programmed death-1 (PD-1) and cytotoxic T-lymphocyte antigen-4 (CTLA-4) on CD4 lymphocytes, and myeloid derived suppressor cells (MDSCs) among the patients with nontuberculous mycobacteria-lung disease (NTM-LD) according to (**A**) positive or negative acid-fast bacilli staining for sputum, and (**B**) initial radiographic findings. The data in the bar charts are mean values and error bars are standard errors. The data were compared using the Mann Whitney U test. Y axis means percentage in (1) lymphocyte population for Treg, PD-1^+^CD4^+^, and CTLA4^+^CD4^+^ cells, and in (2) peripheral blood mononuclear cells for MDSCs. NTM-LD includes *Mycobacterium avium* complex and *M. abscessus* lung disease. FC, fibro-cavitary; NB, nodular bronchiectasis. ns, not statistically significant (*p* > 0.05).

We classified the patients with NTM-LD into AFS-positive (*n* = 23) or AFS-negative (*n* = 46) subgroups, and analyzed their differences to understand the influence of bacteria load. There were no significant differences in age, sex, or BMI between the subgroups. The AFS-positive subgroup had more symptoms of hemoptysis (60.9% vs. 19.6%, *p* = 0.001) and radiographic findings of the FC pattern (43.5% vs. 0%, *p* < 0.001) than the AFS-negative subgroup. With regards to immune regulatory cells, there were higher percentages of Treg cells (17.6% vs. 11.4%, *p* = 0.014) and PD-1^+^CD4^+^ lymphocytes (24.5% vs. 14.8%, *p* = 0.009) in the AFS-positive subgroup than in the AFS-negative subgroup. No significant differences in MDSCs and CTLA-4^+^CD4^+^ T lymphocytes was noticed between the two subgroups (Figure 3A).

### 3.5. Differences between Initial Radiographic Patterns

We compared the patients with NTM-LD with the radiographic FC pattern (*n* = 10) to those with the NB pattern (*n* = 48). There were no significant differences in average age, male gender, or radiographic score between the two groups. The patients with the FC pattern had a higher titer of sputum AFS (3 ± 0.9 vs. 0.6 ± 1.1, *p* < 0.001) and proportion of hemoptysis (80% vs. 27%, *p* = 0.002) than those with the NB pattern.

The percentages of PD-1^+^CD4^+^ lymphocytes (30.9 ± 10.4 vs. 15.6 ± 8.4, *p* = 0.002) and Treg cells (24.4 ± 9.9 vs. 12.4 ± 8.1, *p* = 0.001) were significantly higher in the patients with the FC pattern than in those with the NB pattern (Figure 3B). The percentages of MDSCs and CTLA-4^+^CD4^+^ T lymphocytes were not significantly higher in those with the FC pattern.

### 3.6. Radiographic Progression in NTM-LD and The Predictors

**Figure 4:**
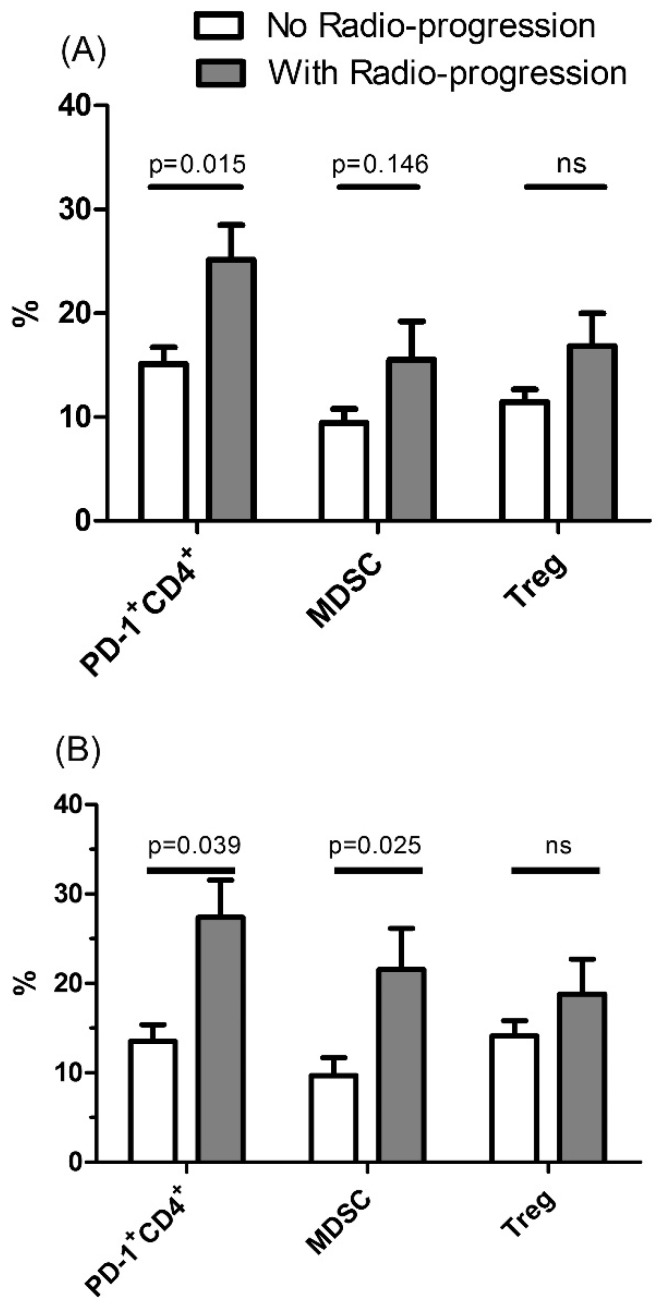
The percentages of programmed death-1^+^ (PD-1^+^) CD4 lymphocytes, myeloid derived suppressor cells (MDSCs), and regulatory T cells (Treg) between the patients with or without radiographic progression. (**A**) Initial data and (**B**) after 2 months of follow-up. Y axis means percentage in (1) lymphocyte population for Treg, PD-1^+^CD4^+^, and CTLA4^+^CD4^+^ cells, and in (2) peripheral blood mononuclear cells for MDSCs. The data in the bar charts and error bars are mean and standard errors, respectively. The data were compared using the Mann Whitney U test. ns, not statistically significant (*p* > 0.05).

Fifty-six patients (39 with MAC-LD and 17 with MAB-LD) underwent follow-up radiography more than 1 year, of whom 14(25%) had radiographic progression. The patients with radiographic progression had higher percentage of PD-1^+^CD4^+^ lymphocytes (25.2 ± 11.5%) than those without progression (15.1 ± 8.6%, *p* = 0.015) (Figure 4A), and the percentages of MDSCs and Treg cells were elevated in those with progression, although the difference did not reach a significant difference (15.5 ± 13.4 vs. 9.4 ± 7.6, *p* = 0.146; and 16.8 ± 16.1 vs. 11.4 ± 7.7, *p* = 0.192, respectively). Of the patients without anti-NTM treatment (*n* = 25), seven (28%) had radiographic progression and the remaining 18 (72%) had stable or resolved radiographic lesions. The percentage of PD-1^+^CD4^+^ lymphocytes (21.5% vs. 13.1%, *p* = 0.041) was still higher in those with radiographic progression than in those without progression.

The crude OR for radiographic progression by the percentage of PD-1^+^CD4^+^ lymphocytes was 1.087 (95% CI: 1.018–1.161, per 1% increment, *p* = 0.013). After adjusting for age and sex by multivariate logistic analysis, the significant predictors of radiographic progression were anti-NTM treatment (OR: 0.136 (0.020–0.914), *p* = 0.040) and the percentage of PD-1^+^CD4^+^ T cells (OR: 1.126 (1.034–1.226), *p* = 0.006).

### 3.7. Follow-up Immune Regulatory Cells for Radiographic Progression in NTM-LD

Two months after enrollment, 21 patients underwent blood sampling to assess follow-up immune profiles. Of them, seven had radiographic progression and their percentages of MDSCs (21.6% vs. 9.6%, *p* = 0.039) and PD-1^+^CD4^+^ lymphocytes (27.4% vs. 13.5%, *p* = 0.025) were higher than in those who were radiographically stable (*n* = 14) (Figure 4B). There was no significant difference in Treg cells (18.8% vs. 14.1%, *p* = 0.315). For the patients who received anti-NTM treatment and blood follow-up (*n* = 13), the percentage of PD-1^+^CD4^+^ lymphocytes was still higher among those with radiographic progression (30.7 ± 8.0% vs. 15.6 ± 7.9%, *p* = 0.049).

## 4. Discussion

In the present study, the PD-1^+^CD4^+^ lymphocytes in the patients with MAC-LD and the MDSCs in those with MAC-LD and MAB-LD were significantly higher compared to the controls. Moreover, the percentages of PD-1^+^CD4^+^ lymphocytes and Treg cells were higher in the NTM-LD patients with the FC radiographic pattern or AFS-positive sputum results than the corresponding groups. With regards to the outcomes, the NTM-LD patients with radiographic progression had higher PD-1^+^CD4^+^ lymphocytes at baseline and 2 months, and this was a significant predictor of radiographic progression after adjustments for age and sex.

PD-1 regulates the activation of T cells by transducing inhibitory signals to T cell receptors [22] and it could represent a marker of induced immune exhaustion [23]. PD-1^+^ lymphocytes have been reported to be increased during a mycobacterial infection, as in the present study, and to be correlated with the attenuation of T cell function and proliferation in tuberculosis and MAC-LD [10,24]. However, few studies have discussed their clinical significance with regards to different subspecies, mycobacteria load, radiographic presentation and progression in NTM-LD.

Among the different subspecies of MAC-LD, *M. intracellulare* and *M. avium* have been reported to be more clinically relevant [25] than *M. chimera*, and possibly to be more virulent. *M. intracellulare* and *M. avium* LD may therefore induce more severe T cell exhaustion through the higher expression of PD-1 on CD4 lymphocytes. In addition, the patients with the FC radiographic pattern, which has been correlated with a higher bacilli load [26] and more severe symptoms, had higher percentages of PD-1^+^CD4^+^ lymphocytes and Treg cells. This suggests that Treg and PD-1 pathway-related immune exhaustion in NTM-LD may be induced by microbiological factors such as the virulence of the strain and high bacilli burden. 

Because the disease course of NTM-LD is usually indolent, the treatment rate is not high [17,19] and radiographic progression is considered for a time-point to commence treatment [19]. In the present study, radiographic progression was as high as 28% for those who did not receive anti-NTM treatment. The best way to predict progression as early as possible is very important. A high percentage of PD-1^+^CD4^+^ lymphocytes correlates with radiographic progression and was a significant predictor. The pathway of PD-1 on lymphocytes has been reported to be induced by chronic MAC infection and to be correlated with immune tolerance [10]. It indicates that a state of host exhaustion may increase the long-term risk of disease progression.

Treg cells regulate the immune response during a host infection and inhibit effector T cells [13]. They have been implicated in immunologic hyporesponsiveness associated with chronic infections including tuberculosis [27]. However, no previous study has investigated the role of Treg cells in NTM-LD. In the present study, the expression of Treg cells was not higher in the patients with NTM-LD, and there was no correlation with radiographic progression. This might be because Treg cells are activated by PD-1 in the chain of immune tolerance and the effect is smaller in NTM-LD. However, the expression of Treg cells increases in the NTM-LD patients with positive AFS or radiographic FC lesions, suggesting that there may be a dose response once a high burden of mycobacterium bacilli has been achieved.

On the other hand, the spectrum of MDSC-related immune suppression is wide, from induction of regulatory T cells [28] to inhibition of T-cell activation and proliferation [29]. The role of MDSCs has not previously been reported in patients with NTM-LD, and the present study showed that MDSCs were associated with increased disease status in both the patients with MAC- and MAB-LD. Two months after enrollment, the percentage of MDSCs was higher in the patients with radiographic progression, and thus MDSC may be a predictor of an early immune response. Further studies are needed to validate this hypothesis.

There are several limitations to this study. First, the sample size was relatively small, especially for the subgroup analysis. Second, we did not match age among the subgroups, although we did adjust for age using multivariate analysis. Third, this study was conducted in Taiwan, and the results may not be generalizable to other countries and ethnicities. The pattern difference of flow cytometry results between this study and previous literature may exist, especially Treg cells, possibly due to the use of different buffer and antibodies [30] as well as cut-off point [31]. Comparison or interpretation should be carefully performed.

## 5. Conclusions

The patients with NTM-LD had a higher expression of MDSCs compared to the controls, and higher expressions of PD-1^+^CD4^+^ lymphocytes were found in the patients with MAC-LD, especially in those with virulent MAC subspecies. In addition, elevated expressions of PD-1^+^CD4^+^ lymphocytes and Treg cells were associated with high NTM burden and the FC radiographic pattern. More importantly, the percentage of PD-1+CD4+ lymphocytes was an independent predictor of radiographic progression, and might be helpful to complement clinical decision making when caring for patients with NTM-LD.

## Figures and Tables

**Table 1:** Clinical characteristics of the participants.

	MAC-LD *n* = 46	MAB-LD *n* = 23	Healthy Subjects *n* = 27
Age (years)	64.5 (15.4)	62.1 (14.0)	55.5 (14.1) *
Male sex	20 (44%)	8 (35%)	15 (56%)
Current smoker	1 (2%)	0	2 (7%)
Body mass index, kg/m^2^	20.0 (3.2)	22.6 (3.7) ^ǂ^	23.1 (2.8) *
Diabetes mellitus	2 (4%)	1 (4%)	2 (7%)
Autoimmune diseases	1 (2%)	1 (4%)	0
Prior TB history	5 (11%)	1 (4%)	0
Symptoms			
Cough	13 (28%)	10 (44%)	-
Dyspnea	22 (48%)	14 (61%)	-
Hemoptysis	15 (33%)	8 (35%)	-
Sputum study within 1 year			
Max. positive AFS	1.1 (1.4)	0.4 (1.1) ^ǂ^	-
No. of positive cultures	3.5 (3.0)	3.0 (1.9)	-
Radiological finding			
CXR score ^#^	4.5 (2.2)	3.0 (2.2) ^ǂ^	-
FC pattern	8 (17%)	2 (9%)	-
NB pattern	32 (70%)	16 (70%)	-

Abbreviations: AFS, acid-fast bacilli staining; CXR, chest X-ray; FC, fibro-cavitary; LD, lung disease; MAC, *Mycobacterium avium* complex; MAB, *Mycobacterium abscessus*; NB, nodular bronchiectasis; TB, tuberculosis data are no. (%) or mean (standard deviation) * and ^ǂ^ indicate *p* < 0.05 between the indicated group and the MAC-LD group, using the chi square test for categorical variables and the Student’s *t* test for numerical variables, ^#^ CXR score was interpreted by a total score from six lung zones that contained three respective scores [21].

## Data Availability

Please contact author for data requests.
